# Influenza Vaccination Among People With Medicare by Race and Ethnicity, Education, and Rurality

**DOI:** 10.1001/jamanetworkopen.2025.4462

**Published:** 2025-04-10

**Authors:** Courtney Gidengil, Amelia Haviland, Katrin Hambarsoomian, Steven Martino, Jacob W. Dembosky, Marc N. Elliott

**Affiliations:** 1 Health Care Department, RAND, Boston, Massachusetts; 2 Heinz College of Information Systems and Public Policy, Carnegie Mellon University, Pittsburgh, Pennsylvania; 3 Behavioral and Policy Sciences, RAND, Pittsburgh, Pennsylvania; 4 Economics, Sociology and Statistics Department, RAND, Santa Monica, California; 5 Health Care Department, RAND, Santa Monica, California

## Abstract

**Importance:**

Influenza vaccination is a safe, effective way to mitigate influenza infection but remains underused by some groups, including older Black and Hispanic adults. There is concern that changing attitudes toward vaccination during the COVID-19 pandemic could decrease influenza vaccination rates.

**Objective:**

To examine national patterns of influenza vaccination among older adults with Medicare, including analyses stratified by race and ethnicity, educational attainment, and rurality.

**Design, Setting, and Participants:**

This retrospective, cross-sectional survey study of responses to the 2019 and 2022 Medicare Consumer Assessment of Healthcare Providers and Systems surveys used cross-sectional analysis for data from a weighted sample of 285 265 community-dwelling Medicare Advantage and Medicare Fee-for-Service enrollees aged 65 years or older living in the 50 US states and Washington, DC, who answered a survey question about influenza immunization.

**Exposures:**

Race and ethnicity, educational attainment, and rurality of survey respondents.

**Main Outcomes and Measures:**

The primary outcome was self-reported influenza vaccination.

**Results:**

The weighted sample combining both years of data for 285 265 individuals included 54.5% women. A total of 4.2% were Asian American, Native Hawaiian, and Other Pacific Islander; 8.0% were Black; 6.9% were Hispanic; and 76.2% were White. Between 2019 and 2022, overall influenza vaccination rates for older adults increased by 0.7 percentage points (95% CI, 0.2-1.1 percentage points), from 76.3% to 77.0%. Whereas influenza vaccination rates for Asian American, Native Hawaiian, and Other Pacific Islander older adults (from 81.2% to 83.1%) and White older adults (from 77.7% to 77.9%) were essentially unchanged between 2019 and 2022, they increased by 3.8 percentage points (95% CI, 1.8-5.7 percentage points) for Black older adults (from 66.9% to 70.7%) and 2.3 percentage points (95% CI, 0.5-4.0 percentage points) for Hispanic older adults (from 72.7% to 75.0%). Black and Hispanic older adults living in rural areas had the largest increases during this period (Black, 7.0 percentage points [95% CI, 0.3-13.8 percentage points]; Hispanic, 8.2 percentage points [95% CI, 0.8-15.5 percentage points]), while White older adults with lower educational attainment and/or living in rural areas had decreases in vaccination rates (White with lower educational attainment, –1.9 percentage points [95% CI, −2.8 to −1.0 percentage points]; White living in rural areas, –2.0 percentage points [95% CI, −3.2 to −0.8 percentage points]; White with lower educational attainment and living in rural areas, –3.7 percentage points [95% CI, −5.5 to −1.9 percentage points]).

**Conclusions and Relevance:**

In this cross-sectional survey study, although overall influenza vaccination rates changed little from 2019 to 2022, they increased substantially for Black and Hispanic older adults, particularly those in rural areas, and decreased for some groups of White older adults. Determining the reasons for these divergent changes in influenza vaccination rates is a high priority for future research.

## Key Points

**Question:**

How did influenza vaccination rates among people aged 65 years or older change during the COVID-19 pandemic?

**Findings:**

This cross-sectional survey study examined patterns of influenza vaccination among a weighted sample of 285 265 people with Medicare aged 65 years or older using data from the Medicare Consumer Assessment of Healthcare Providers and Systems survey. White older adults with lower educational attainment and/or in rural areas had a decrease of approximately 2.0 percentage points, while Black and Hispanic older adults living in rural areas had the largest increases (7.0 and 8.2 percentage points, respectively).

**Meaning:**

These findings indicate increases for populations with historically low vaccination.

## Teaser

This cross-sectional survey study assesses national patterns of influenza vaccination during the COVID-19 pandemic among older adults with Medicare, including analyses stratified by race and ethnicity, educational attainment, and rurality.

## Introduction

Influenza infection is associated with significant morbidity and mortality in the US. Estimates from the Centers for Disease Control and Prevention (CDC) for the 2022-2023 season alone indicate that there were 14 million (95% CI, 12 million to 21 million) medical visits for influenza, 300 000 (95% CI, 298 000-614 000) influenza-related hospitalizations, and 21 000 (95% CI, 16 000-41 000) influenza deaths.^1^ That burden is most concentrated among people aged 65 years or older (hereafter, *older adults*), who experience notably higher rates of influenza-related hospitalizations and deaths, as well as influenza-related complications of chronic diseases.

In addition, being American Indian or Alaska Native, Black, or Hispanic is associated with a higher risk of influenza-related hospitalizations. Between 2009-2010 and 2021-2022, age-adjusted influenza hospitalization rates were significantly higher among non-Hispanic American Indian or Alaska Native adults (54.6 hospitalizations per 100 000 population; risk ratio [RR], 1.27 [95% CI, 1.19-1.36]), Black adults (78.2 hospitalizations per 100 000 population; RR, 1.82 [95% CI, 1.79-1.84]), and Hispanic adults (50.3 hospitalizations per 100 000 population; RR, 1.17 [95% CI, 1.14-1.19]), compared with the rate among White adults (43.0 hospitalizations per 100 000 population).^2^

Influenza vaccination is a safe and effective way to mitigate the burden of influenza infection. Among older adults, influenza vaccination significantly reduces hospitalizations and other complications due to influenza.^3^ Although influenza vaccination rates have slowly increased over the past 10 years and are highest among older adults, vaccination remains underused, particularly for American Indian or Alaska Native, Black, and Hispanic adults. When considering all adults, influenza vaccination during the 2021-2022 season was lower among Hispanic (37.9%), American Indian or Alaska Native (40.9%), and Black (42.0%) adults than among White (53.9%) and Asian American (54.2%) adults^4^; this pattern continued through the 2022-2023 season^5^ and has been consistently observed since the 2010-2011 season.^2^ Disparities in vaccination rates by race and ethnicity were present even among adults of all ages who reported having medical insurance, a personal health care professional, and a routine medical checkup in the past year.^2^

There has also been concern that the COVID-19 pandemic and related safety concerns about the COVID-19 vaccine could promote general mistrust of vaccines among some groups, potentially reducing influenza vaccination rates.^6^ One study suggests that while influenza vaccine uptake remained relatively stable during the first influenza season of the COVID-19 pandemic, it decreased within states in the bottom 2 quartiles of COVID-19 vaccine uptake and increased within states in the top 2 quartiles.^7^ In this same study, influenza vaccination rates were found to have remained relatively stable among older adults, although analyses by race and ethnicity were not reported. Mistrust in COVID-19 vaccines and the associated unwillingness to be vaccinated are particularly pronounced among people with lower educational attainment and those who live in rural areas,^8^ yet little is known about the variation in influenza vaccination during the COVID-19 pandemic among these subgroups at a national level.

Understanding national trends in influenza vaccination for older adults during the years surrounding a sentinel event such as the COVID-19 pandemic is critical to informing subsequent investments in information campaigns and efforts to increase access for this population. Although the CDC publishes robust influenza vaccination rates by race and ethnicity and age group based on the Behavioral Risk Factor Surveillance System (BRFSS), results accounting for both factors are hampered by limited sample sizes for most racial and ethnic groups. In addition, assessing interactions involving other key characteristics, such as educational attainment and rurality, which are not available in the CDC’s data from the BRFSS, is needed to better understand the observed patterns and shape policy responses.

In this study, we use national data from the Medicare Consumer Assessment of Healthcare Providers and Systems (MCAHPS) survey to examine patterns of influenza vaccination among older adults in the US before vs late in the COVID-19 pandemic. The larger sample size and richer data allow for the investigation of national patterns by race and ethnicity, educational attainment, and rurality.

## Methods

### Sample and Procedures

The study used 2019 and 2022 MCAHPS data, which are nationally representative of the community-dwelling Medicare population.^9^ Our analysis was limited to 515 985 Medicare Advantage and Medicare Fee-for-Service enrollees aged 65 years or older living in the 50 US states or Washington, DC, who responded to a survey question about influenza vaccination. Five percent of respondents did not respond to the influenza vaccination survey question (or selected the “Don’t Know” response option). Response rates for this sample were 38.2% in 2019 and 35.6% in 2022. This study follows the Strengthening the Reporting of Observational Studies in Epidemiology (STROBE) reporting guideline for cross-sectional studies. This study was reviewed and approved by the RAND Human Subjects Protection Committee. Participation in the survey was voluntary; receipt of a completed survey was taken as an indication of informed consent.

### Outcomes, Covariates, and Other Measures

Influenza vaccination status, the outcome variable in our analysis, was self-reported via an item asking about receiving an influenza vaccine (“flu shot”) in the previous year (with response options of “Yes,” “No,” and “Don’t Know”). Race and ethnicity were self-reported in response to separate survey questions about Hispanic ethnicity and race. Respondents who endorsed Hispanic ethnicity were classified as Hispanic. Those not endorsing Hispanic ethnicity were classified as American Indian or Alaska Native; Asian American, Native Hawaiian, or Other Pacific Islander; Black; multiracial (if >1 race was reported); White; or unknown. Most analyses were restricted to the 4 largest race and ethnicity groups: Asian American, Native Hawaiian, and Other Pacific Islander; Black; Hispanic; and White, although all groups are included in the totals. Data on educational attainment were also self-reported and used to classify respondents into 2 categories: lower educational attainment (having at most a high school degree or General Educational Development certificate) and higher educational attainment (any college education or greater). Classification of respondents by place of residence was based on the zip code of their mailing address and the associated Core-Based Statistical Area. Respondents living outside of a Core-Based Statistical Area and in micropolitan statistical areas (a core urban area of ≥10 000 but <50 000 residents) were classified as rural; respondents living in metropolitan divisions and metropolitan statistical areas (a core or urban area of ≥50 000 residents) were classified as urban.

### Statistical Analysis

We produced influenza vaccination rate estimates, 95% CIs, and *P* values for change over time overall, by race and ethnicity, and for the 4 largest race and ethnicity groups (Asian American, Native Hawaiian, and Other Pacific Islander; Black; Hispanic; and White) by rurality, educational attainment, and the 4-way combination of these 2 classifications. The 95% CIs and hypothesis tests were 2-sided; *P* < .05 was considered significant. *P* values between *P* = .05 and *P* = .10 are reported and described as suggestive of a difference. Estimates came from linear regression models for each racial and ethnic group. The linear model allows coefficients to be interpreted as changes in the proportion of people vaccinated. At the sample sizes used here, the assumption that SEs of regression coefficients are normally distributed is satisfied.^10^ Model 1 had 1 independent variable, a 2022 indicator. Model 2 added an indicator for lower educational attainment and its interaction with the 2022 indicator. Model 3 added to model 1 an indicator for higher educational attainment and its interaction with the 2022 indicator. Model 4 included all 3 indicators, their 2-way interactions, and the one 3-way interaction. Full model results appear in eTables 1 to 4 in Supplement 1. To address concerns about the familywise error rate, omnibus tests on 3 groups of interactions from a second set of models were performed. Details and results appear in eAppendix 1 and eTable 5 in Supplement 1.

Person-level poststratification weights were used for all analyses to account for sample design and nonresponse.^11,12,13^ Detailed administrative data on people with Medicare allowed the weights to exactly match the community-dwelling older adult Medicare population on numerous demographic proportions.^9,13^ We used Stata, version 18 software (StataCorp LLC) to produce all results and figures.

## Results

Combining both years of data, the weighted sample of 285 265 individuals included 54.5% women and 45.5% men. A total of 4.2% were Asian American, Native Hawaiian, and Other Pacific Islander; 8.0% were Black; 6.9% were Hispanic; and 76.2% were White; 97.5% of responses were in English, 2.4% in Spanish, and 0.1% in other languages. In 2022, urban residence ranged from 96.9% (96.6% in 2019) for Asian American, Native Hawaiian, and Other Pacific Islander adults to 80.6% (80.0% in 2019) for White adults (Table; eTable 6 in Supplement 1). Higher educational attainment in 2022 ranged from 65.9% (62.9% in 2019) for Asian American, Native Hawaiian, and Other Pacific Islander adults to 36.9% (33.5% in 2019) for Hispanic adults.

**Table.** Sample Sizes, Educational Attainment, and Rural Residence Rates by Race and Ethnicity^a^

Characteristic	All^b^	Asian American, Native Hawaiian and Other Pacific Islander	Black	Hispanic	White
**2019 Survey data**					
Sample size, No. (%)	230 720 (100)	8538 (3.8)	19 535 (7.8)	19 436 (6.5)	172 365 (76.9)
% Higher educational attainment	60.3	62.9	48.1	33.5	64.0
% Lower educational attainment	37.2	35.3	48.5	60.3	34.4
% Missing educational attainment	2.5	1.8	3.3	6.2	1.7
% Urban	82.1	96.6	89.2	92.2	80.0
% Rural	17.9	3.4	10.8	7.8	20.0
**2022 Survey data**					
Sample size, No. (%)	285 265 (100)	11 349 (4.5)	22 498 (8.2)	20 901 (7.2)	217 423 (75.4)
% Higher educational attainment	62.4	65.9	52.4	36.9	65.8
% Lower educational attainment	35.9	32.5	45.8	59.1	33.0
% Missing educational attainment	1.8	1.7	1.9	3.9	1.2
% Urban	83.1	96.9	90.2	93.3	80.6
% Rural	17.0	3.1	9.8	6.7	19.4

^a^ All percentages are weighted; all numbers are unweighted (see eTable 6 in Supplement 1 for all sample sizes and unweighted percentages).

^b^ Includes all 7 race and ethnicity groups.

**Figure 1.**
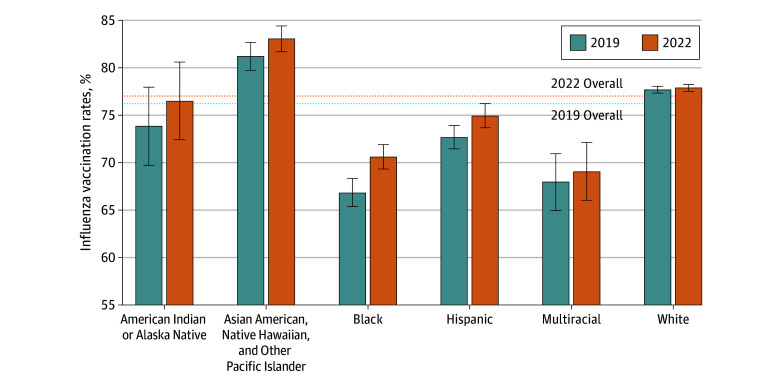
National Influenza Vaccination Rates by Race and Ethnicity, 2019 and 2022 Error bars indicate 95% CIs.

Prior to 2019, overall influenza vaccination rates had been increasing for older adults at least since 2010, with the largest increases observed among Black and Hispanic adults, resulting in less pronounced differences than were observed before that time (eAppendix 2 in Supplement 1). Between 2019 (the season just prior to the COVID-19 pandemic) and 2022 (the third year of the COVID-19 pandemic), overall influenza vaccination rates for older adults increased slightly by 0.7 percentage points (95% CI, 0.2-1.1 percentage points) from 76.3% to 77.0% (Figure 1). Although there was no clinically significant change across the entire older adult population, when examining changes by race and ethnicity, there was an increase of 3.8 percentage points (95% CI, 1.8-5.7 percentage points) for Black older adults (from 66.9% to 70.7%) and 2.3 percentage points (95% CI, 0.5-4.0 percentage points) for Hispanic older adults (from 72.7% to 75.0%). By contrast, there was no evidence of significant change for Asian American, Native Hawaiian, and Other Pacific Islander older adults (increase of 1.9 percentage points [95% CI, −0.1 to 3.9 percentage points] from 81.2% to 83.1%) or White older adults (increase of 0.2 percentage points [95% CI, −0.3 to 0.7 percentage points] from 77.7% to 77.9%).

**Figure 2.**
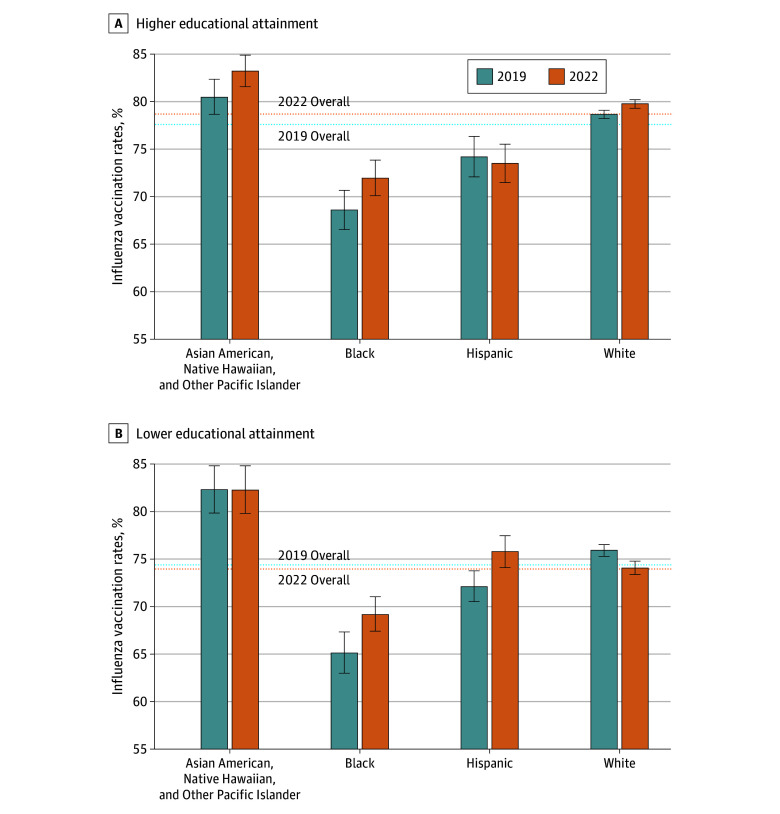
National Influenza Vaccination Rate by Race and Ethnicity and Educational Attainment, 2019 and 2022 A, Higher educational attainment (any college or higher). B, Lower educational attainment (high school degree or General Educational Development certificate or lower). Error bars indicate 95% CIs.

**Figure 3.**
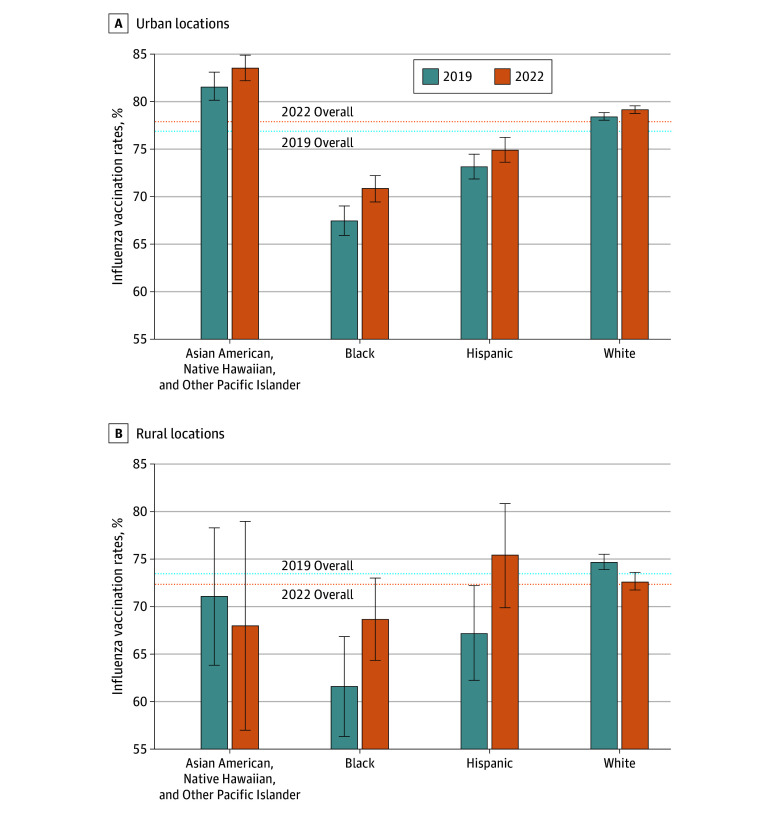
National Influenza Vaccination Rates by Race and Ethnicity and Rurality, 2019 and 2022 Error bars indicate 95% CIs.

For Asian American, Native Hawaiian, and Other Pacific Islander older adults, there was an increase in influenza vaccination rates only for those with higher educational attainment (increase of 2.8 percentage points [95% CI, 0.3-5.2 percentage points]), but the change was not statistically significant, while no significant change was detected for those with lower educational attainment (Figure 2). For Asian American, Native Hawaiian, and Other Pacific Islander older adults in urban areas, vaccination rates increased by 2.0 percentage points (95% CI, −0.03 to 3.99 percentage points); estimates for rural areas are limited by small sample sizes (Figure 3). The largest estimated change among Asian American, Native Hawaiian, and Other Pacific Islander older adults was for those with higher educational attainment in urban areas (increase of 3.0 percentage points [95% CI, 0.5-5.5 percentage points]).

For Black older adults, there were similar increases in influenza vaccination rates among those with higher and lower educational attainment (Figure 2). Those with higher educational attainment had an increase of 3.4 percentage points (95% CI, 0.6-6.2 percentage points), and those with lower educational attainment had an increase of 4.1 percentage points (95% CI, 1.3-6.9 percentage points). Black older adults in rural areas had an increase of 7.0 percentage points (95% CI, 0.3-13.8 percentage points), while those in urban areas had an increase of 3.4 percentage points (95% CI, 1.3-5.4 percentage points) (Figure 3). Neither difference in increases, between higher and lower education or between urban and rural, was statistically significant.

For Hispanic older adults, there was an increase in influenza vaccination rates among those with lower educational attainment (increase of 3.7 percentage points [95% CI, 1.4-6.0 percentage points]); no significant change was detected for those with higher educational attainment (difference in change between higher and lower education is statistically significant) (Figure 2). This increase appears to have been concentrated among rural Hispanic older adults, where there was an increase of 8.2 percentage points (95% CI, 0.8-15.5 percentage points), compared with those in urban areas (increase of 1.8 percentage points [95% CI, −0.1 to 3.6 percentage points]; *P* = .10) (Figure 3). When examining changes only among Hispanic older adults with lower educational attainment, increases in influenza vaccination rates were estimated to be 3 times higher among those who lived in rural areas (increase of 11.0 percentage points [95% CI, 1.8-20.1 percentage points]) compared with those who lived in urban areas (increase of 3.0 percentage points [95% CI, 0.6-5.4 percentage points]; *P* = .10).

For White older adults, there was a small increase in influenza vaccination rates for those with higher educational attainment (increase of 1.1 percentage points [95% CI, 0.5-1.7 percentage points]) but a decrease for those with lower educational attainment (decrease of 1.9 percentage points [95% CI, −2.8 to −1.0 percentage points]; *P* < .001) (Figure 2). There were also differences by rurality, with a decrease in influenza vaccination rates among White older adults in rural areas (decrease of 2.0 percentage points [95% CI, −3.2 to −0.8 percentage points]) and a slight increase for those in urban areas (increase of 0.7 percentage points [95% CI, 0.2-1.3 percentage points]; *P* < .001) (Figure 3). When considering both educational attainment and rurality among White older adults, an increase in influenza vaccination rates was detected only for those who both lived in urban areas and had higher educational attainment (increase of 1.5 percentage points [95% CI, 0.8-2.1 percentage points]; difference from those in rural areas who had higher educational attainment and difference from those who lived in urban areas who had lower educational attainment are both statistically significant). By contrast, there was a decrease for White older adults with lower educational attainment for both those in rural areas (decrease of 3.7 percentage points [95% CI, −5.5 to −1.9 percentage points]) and those in urban areas (decrease of 1.2 percentage points [95% CI, −2.3 to −0.2 percentage points]; *P* = .02 for difference).

## Discussion

In this analysis of MCAHPS data, we found that the overall influenza vaccination rate for US adults aged 65 years or older was largely unchanged from 2019 to 2022, which coincides with the timing of the COVID-19 pandemic. However, we identified significant changes when examining changes by race and ethnicity, educational attainment, and rurality. Rural Black and Hispanic older adults had the largest estimated increases during this period, at 7.0 percentage points for Black older adults and 8.2 percentage points for Hispanic older adults, while White older adults with lower educational attainment and/or living in rural areas had the largest estimated decreases in influenza vaccination rates at approximately 2.0 percentage points.

These findings are encouraging for populations that have been historically more difficult to reach with interventions. We can only speculate about the reasons for the substantial increases in influenza vaccination rates seen among rural Black and Hispanic older adults, the modest increases among urban Black older adults and Hispanic older adults with lower educational attainment, and the decreases seen among White older adults with lower educational attainment and/or living in rural areas. One possibility is that interventions intended to increase underserved populations’ access to COVID-19 vaccines had a spillover effect on influenza vaccination rates. This hypothesis is supported by data from other studies indicating higher COVID-19 vaccination rates for Black adults in both urban and rural areas compared with White adults. For example, in 1 study of rates of uptake of the first dose of the COVID-19 vaccine among US veterans, Asian American, Native Hawaiian, and Other Pacific Islander; Black; and Hispanic veterans in urban areas were more likely than their White counterparts to receive a first COVID-19 vaccination; rural Black adults were 6% more likely to receive a first COVID-19 vaccination than urban White adults.^14^ The authors concluded that a proactive approach in a primary care–focused health care system to reduce barriers and address concerns may have increased vaccination rates among people from racial and ethnic minority groups. Another study found that rural White adults reported significantly less willingness to be vaccinated for COVID-19 than their nonrural White counterparts, whereas little difference was seen between rural and nonrural Black and Hispanic adults.^15^

In contrast with older adults in other racial and ethnic groups, White older adults with lower educational attainment and/or living in rural areas were less likely to receive the influenza vaccination in 2022 compared with 2019. Any decrease in influenza vaccination among older adults is concerning and contrary to the positive trend in annual influenza vaccination over the prior decade. Again, our data are observational and only allow us to speculate, but it is possible that a tendency to avoid COVID-19 vaccines extended to vaccines more generally, including influenza vaccines. This hypothesis is supported by 1 study that found a correlation between widespread availability of COVID-19 vaccines (due to low uptake) and decrease in influenza vaccination rates.^7^ If this is indeed the cause, an important question is why a tendency to avoid COVID-19 vaccines would translate into reduced influenza vaccination rates only for White older adults with lower educational attainment and/or living in rural areas.

Despite substantial increases in vaccination rates for populations with historically low vaccination, there is still significant variation in influenza vaccination rates among older adults. Black older adults had the largest influenza vaccination gap compared with Asian American, Native Hawaiian, and Other Pacific Islander older adults, who had the highest rate (70.7% vs 83.1%), which highlights the importance of continuing to invest in efforts to reach immunization goals. If the increases seen in influenza vaccination rates for most racial and ethnic groups were indeed due to the efforts to provide broad access to COVID-19 vaccination (eg, making vaccines available free of charge at varied and nontraditional locations),^16,17^ we may very well see losses of these prior gains in the absence of ongoing investment. Differences in receipt of COVID-19 booster doses may be associated with such a decrease in influenza vaccination rates among Black and Hispanic populations.^18,19^

### Limitations

This study has some limitations. Although our sample sizes are large, most analyses were limited to the 4 largest racial and ethnic groups, and further study is needed to determine educational attainment and urban and rural influenza vaccination differences for smaller groups, including American Indian or Alaska Native and multiracial older adults. Influenza vaccination status was determined by self-report on the survey; however, multiple studies have previously established the validity of this method for determining influenza vaccination status.^20,21,22^ Finally, we did not have access to individual-level data on COVID-19 vaccination status. An important contribution of future work will be to explore whether those not receiving COVID-19 vaccines were also less likely to receive an influenza vaccine.

The influenza vaccination rate for older adults in our study of 2022 data for the 2021-2022 flu season was 77.0% (95% CI, 76.6%-77.3%), higher than the CDC’s BRFSS estimates for 2021-2022 (73.9% [95% CI, 73.1%-74.7%]).^4^ One reason for this difference may be that our study population is insured and therefore may be more likely to be exposed to opportunities for influenza vaccination due to increased access to health care. However, given that 97% of older adults in the US are covered under Medicare, insurance coverage alone is unlikely to explain the differences. However, our sample size (285 265 older adult respondents) is more than twice the size of the corresponding BRFSS sample (108 726), which allows for more precise estimates, particularly by race and ethnicity, educational attainment, and rurality.

## Conclusions

In this cross-sectional survey study, we analyzed influenza vaccination rates in a large sample of adults aged 65 years or older with Medicare. Although overall influenza vaccination rates did not change substantially from 2019 to 2022, there were substantial increases for Black and Hispanic older adult, populations with historically low vaccination, while White older adults with lower educational attainment and/or living in rural areas had decreased rates of vaccination. The increases observed among Black and Hispanic older adults were more pronounced in rural than in urban areas. Determining the reasons for these divergent changes is a high priority for future studies. However, these descriptive findings are important to policymakers as they make decisions about how to invest in interventions that could influence influenza vaccination rates for populations with lower rates of uptake.

## Notes

Supplement 1. **eAppendix 1.** Supplemental Text on Omnibus Tests **eAppendix 2.** Supplemental Text on Changes in Vaccination Rates by Race and Ethnicity From 2010 to 2019 **eTable 1.** National Influenza Vaccination Rates by Race and Ethnicity, 2019-2022 **eTable 2.** National Influenza Immunization Rates by Race and Ethnicity and Educational Attainment, 2019-2022 **eTable 3.** National Influenza Immunization Rates by Race and Ethnicity and Rurality, 2019-2022 **eTable 4.** Model 4 Results: Linear Regression Model Estimates Predicting Influenza Vaccination by Race and Ethnicity, Educational Attainment, and Rurality, 2019-2022 **eTable 5.** Linear Regression Model Estimates Predicting Influenza Immunization with Omnibus Tests for Race and Ethnicity and Its Interactions **eTable 6.** Sample Sizes, Educational Attainment, and Rural Residence Rates by Race and Ethnicity, Weighted and Unweighted

Supplement 2.
Data Sharing Statement
