# Supplementary material for: Influenza Vaccination Among People With Medicare by Race and Ethnicity, Education, and Rurality
Source: JAMA Netw Open. 2025 Apr 10;8(4):e254462. doi: 10.1001/jamanetworkopen.2025.4462 (PMC11986769; doi:10.1001/jamanetworkopen.2025.4462)
Supplement: Supplement 1. — eAppendix 1. Supplemental Text on Omnibus Tests eAppendix 2. Supplemental Text on Changes in Vaccination Rates by Race and Ethnicity From 2010 to 2019 eTable 1. National Influenza Vaccination Rates by Race and Ethnicity, 2019-2022 eTable 2. National Influenza Immunization Rates by Race and Ethnicity and Educational Attainment, 2019-2022 eTable 3. National Influenza Immunization Rates by Race and Ethnicity and Rurality, 2019-2022 eTable 4. Model 4 Results: Linear Regression Model Estimates Predicting Influenza Vaccination by Race and Ethnicity, Educational Attainment, and Rurality, 2019-2022 eTable 5. Linear Regression Model Estimates Predicting Influenza Immunization with Omnibus Tests for Race and Ethnicity and Its Interactions eTable 6. Sample Sizes, Educational Attainment, and Rural Residence Rates by Race and Ethnicity, Weighted and Unweighted [file jamanetwopen-e254462-s001.pdf]

Supplementary Online Content

Gidengil C, Haviland A, Hambarsoomian K, Martino S, Dembosky JW, Elliott MN. Influenza vaccination among people with Medicare by race and ethnicity, education, and rurality. *JAMA Netw Open.* 2025;8(4):e254462.
doi:10.1001/jamanetworkopen.2025.4462

eAppendix 1. Supplemental Text on Omnibus Tests
eAppendix 2. Supplemental Text on Changes in Vaccination Rates by Race and Ethnicity From 2010 to 2019
eTable 1. National Influenza Vaccination Rates by Race and Ethnicity, 2019-2022
eTable 2. National Influenza Immunization Rates by Race and Ethnicity and Educational Attainment, 2019-2022
eTable 3. National Influenza Immunization Rates by Race and Ethnicity and Rurality, 2019-2022
eTable 4. Model 4 Results: Linear Regression Model Estimates Predicting Influenza Vaccination by Race and Ethnicity, Educational Attainment, and Rurality, 2019-2022
eTable 5. Linear Regression Model Estimates Predicting Influenza Immunization with Omnibus Tests for Race and Ethnicity and Its Interactions
eTable 6. Sample Sizes, Educational Attainment, and Rural Residence Rates by Race and Ethnicity, Weighted and Unweighted

This supplementary material has been provided by the authors to give readers additional information about their work.

© 2025 Gidengil C et al. *JAMA Network Open.*

eAppendix 1. Supplemental Text on Omnibus Tests

To address concerns about family-wise error rate, a second set of models were run where omnibus tests on the three groups of interactions from the main models were performed. Observations from the main models were pooled with indicators for race-and-ethnicity and their interactions with (a) the 2022 indicator (b) the 2022 and higher education indicators and (c) the 2022 and rurality indicators.

As can be seen in eTable 5, all three omnibus tests, run to address concerns about family-wise error rates, were highly statistically significant, $p < 0.001$, $p < 0.001$, $p = 0.01$ respectively.

© 2025 Gidengil C et al. *JAMA Network Open.*

eAppendix 2. Supplemental Text on Changes in Vaccination Rates by Race and Ethnicity From 2010 to 2019

Between 2010 and 2019, the influenza vaccination rate for people 65 years of age and older in the U.S. increased from 69% to 76%. In 2010, there was substantial variation in influenza vaccination by race-and-ethnicity among the four largest groups: 75% for AANHPI, 71% for White, 64% for Hispanic, and 54% for Black adults aged 65 years and older. There were increases in vaccination rates for all four groups, with larger increases for Hispanic (increase of 9 percentage points) and Black (increase of 13 percentage points) than AANHPI (increase of 6 percentage points) and non-Hispanic White (increase of 7 percentage points) people. These changes resulted in a reduction in influenza vaccination differences by 2019. Relative to the overall influenza vaccination rate, AANHPI were 5 percentage points higher, non-Hispanic White 1 percentage point higher, Hispanic 4 percentage points lower, and Black 9 percentage points lower.

© 2025 Gidengil C et al. *JAMA Network Open.*

eTable 1. National Influenza Vaccination Rates by Race and Ethnicity, 2019-2022

Race-and-ethnicity	Year	Estimate	Standard Error	95% CI, Lower	95% CI, Upper	p-value
AI/AN	2019	73.86	2.10	69.75	77.97	
AI/AN	2022	76.53	2.08	72.45	80.62	
AI/AN	2019-2022 Change	2.67	2.96	-3.12	8.47	0.366
AA & NHPI	2019	81.20	0.75	79.74	82.66	
AA & NHPI	2022	83.05	0.69	81.69	84.41	
AA & NHPI	2019-2022 Change	1.85	1.02	-0.15	3.85	0.069
Black	2019	66.89	0.75	65.43	68.36	
Black	2022	70.67	0.66	69.37	71.96	
Black	2019-2022 Change	3.77	1.00	1.82	5.73	0.000
Hispanic	2019	72.72	0.63	71.49	73.95	
Hispanic	2022	74.97	0.65	73.70	76.24	
Hispanic	2019-2022 Change	2.25	0.90	0.48	4.02	0.013
Multiracial	2019	67.98	1.54	64.96	70.99	
Multiracial	2022	69.12	1.55	66.09	72.15	
Multiracial	2019-2022 Change	1.14	2.18	-3.13	5.42	0.600
White	2019	77.69	0.18	77.34	78.03	
White	2022	77.89	0.19	77.53	78.26	
White	2019-2022 Change	0.21	0.25	-0.29	0.71	0.415

AANHPI = Asian American and Native Hawaiian or other Pacific Islander.

© 2025 Gidengil C et al. *JAMA Network Open.*

eTable 2. National Influenza Immunization Rates by Race and Ethnicity and Educational Attainment, 2019-2022

Race-and-Ethnicity	Education Category/Year or Change	Estimate	Standard Error	95% CI, Lower	95% CI, Upper	p-value
AA & NHPI	Any College or Higher Educational Attainment, 2019	80.64	0.95	78.78	82.49	
AA & NHPI	Any College or Higher Educational Attainment, 2022	83.39	0.83	81.76	85.02	
AA & NHPI	High School Degree/GED or lower Educational Attainment, 2019	82.34	1.25	79.89	84.78	
AA & NHPI	High School Degree/GED or lower Educational Attainment, 2022	82.30	1.29	79.77	84.82	
AA & NHPI	Any College or Higher Educational Attainment, 2019-2022 change	2.75	1.26	0.29	5.22	0.029
AA & NHPI	High School Degree/GED or lower Educational Attainment, 2019-2022 change	-0.04	1.79	-3.55	3.47	0.982
AA & NHPI	Differential Change from 2019 to 2022	2.79	2.19	-1.50	7.08	0.202
Black	Any College or Higher Educational Attainment, 2019	68.67	1.05	66.62	70.73	
Black	Any College or Higher Educational Attainment, 2022	72.04	0.96	70.15	73.93	
Black	High School Degree/GED or lower Educational Attainment, 2019	65.10	1.10	62.94	67.26	
Black	High School Degree/GED or lower Educational Attainment, 2022	69.19	0.92	67.38	71.00	
Black	Any College or Higher Educational Attainment, 2019-2022 change	3.37	1.42	0.58	6.16	0.018
Black	High School Degree/GED or lower Educational Attainment, 2019-2022 change	4.09	1.44	1.28	6.91	0.004
Black	Differential Change from 2019 to 2022	-0.72	2.02	-4.69	3.24	0.722
Hispanic	Any College or Higher Educational Attainment, 2019	74.31	1.08	72.18	76.43	

© 2025 Gidengil C et al. *JAMA Network Open.*

Hispanic	Any College or Higher Educational Attainment, 2022	73.60	1.04	71.57	75.63	
Hispanic	High School Degree/GED or lower Educational Attainment, 2019	72.11	0.81	70.52	73.69	
Hispanic	High School Degree/GED or lower Educational Attainment, 2022	75.77	0.86	74.09	77.45	
Hispanic	Any College or Higher Educational Attainment, 2019-2022 change	-0.70	1.50	-3.65	2.24	0.638
Hispanic	High School Degree/GED or lower Educational Attainment, 2019-2022 change	3.66	1.18	1.35	5.98	0.002
Hispanic	Differential Change from 2019 to 2022	-4.37	1.91	-8.11	-0.63	0.022
White	Any College or Higher Educational Attainment, 2019	78.78	0.22	78.35	79.20	
White	Any College or Higher Educational Attainment, 2022	79.90	0.22	79.48	80.33	
White	High School Degree/GED or lower Educational Attainment, 2019	75.89	0.30	75.30	76.48	
White	High School Degree/GED or lower Educational Attainment, 2022	74.03	0.35	73.34	74.72	
White	Any College or Higher Educational Attainment, 2019-2022 change	1.13	0.31	0.53	1.73	0.000
White	High School Degree/GED or lower Educational Attainment, 2019-2022 change	-1.86	0.46	-2.77	-0.95	0.000
White	Differential Change from 2019 to 2022	2.98	0.56	1.89	4.07	0.000

AANHPI = Asian American and Native Hawaiian or other Pacific Islander.

Differential Change from 2019 to 2022 = (Any College or Higher Educational Attainment, 2022 - 2019) - (High School Degree/GED or lower Educational Attainment, 2022-2019)

© 2025 Gidengil C et al. *JAMA Network Open.*

eTable 3. National Influenza Immunization Rates by Race and Ethnicity and Rurality, 2019-2022

Race-and-Ethnicity	Rurality/Year or Change	Estimate	Standard Error	95% CI, Lower	95% CI, Upper	p-value
AA & NHPI	Urban Locations, 2019	81.56	0.76	80.07	83.05	
AA & NHPI	Urban Locations, 2022	83.54	0.69	82.19	84.88	
AA & NHPI	Rural Locations, 2019	71.05	3.67	63.86	78.24	
AA & NHPI	Rural Locations, 2022	67.98	5.60	57.02	78.95	
AA & NHPI	Urban Locations, 2019-2022 change	1.98	1.02	-0.03	3.99	0.053
AA & NHPI	Rural Locations, 2019-2022 change	-3.06	6.69	-16.18	10.05	0.647
AA & NHPI	Differential Change from 2019 to 2022	-5.05	6.77	-18.31	8.22	0.456
Black	Urban Locations, 2019	67.53	0.77	66.02	69.04	
Black	Urban Locations, 2022	70.88	0.69	69.52	72.25	
Black	Rural Locations, 2019	61.61	2.64	56.44	66.79	
Black	Rural Locations, 2022	68.66	2.21	64.33	72.99	
Black	Urban Locations, 2019-2022 change	3.35	1.04	1.32	5.38	0.001
Black	Rural Locations, 2019-2022 change	7.04	3.44	0.29	13.79	0.041
Black	Differential Change from 2019 to 2022	3.69	3.60	-3.36	10.74	0.305
Hispanic	Urban Locations, 2019	73.19	0.65	71.92	74.46	
Hispanic	Urban Locations, 2022	74.94	0.66	73.64	76.24	
Hispanic	Rural Locations, 2019	67.21	2.52	62.27	72.15	
Hispanic	Rural Locations, 2022	75.37	2.78	69.91	80.82	
Hispanic	Urban Locations, 2019-2022 change	1.75	0.93	-0.06	3.57	0.059
Hispanic	Rural Locations, 2019-2022 change	8.15	3.75	0.80	15.51	0.030
Hispanic	Differential Change from 2019 to 2022	6.40	3.87	-1.18	13.98	0.098
White	Urban Locations, 2019	78.44	0.19	78.06	78.82	
White	Urban Locations, 2022	79.15	0.20	78.76	79.55	
White	Rural Locations, 2019	74.67	0.40	73.89	75.45	
White	Rural Locations, 2022	72.64	0.46	71.74	73.54	
White	Urban Locations, 2019-2022 change	0.72	0.28	0.17	1.26	0.010
White	Rural Locations, 2019-2022 change	-2.03	0.61	-3.22	-0.83	0.001
White	Differential Change from 2019 to 2022	-2.75	0.67	-4.06	-1.43	0.000

© 2025 Gidengil C et al. *JAMA Network Open.*

AANHPI = Asian American and Native Hawaiian or other Pacific Islander.

Differential Change from 2019 to 2022 = (Urban Locations, 2022 - 2019) - (Rural Locations, 2022-2019)

© 2025 Gidengil C et al. *JAMA Network Open.*

eTable 4. Model 4 Results: Linear Regression Model Estimates Predicting Influenza Vaccination by Race and Ethnicity, Educational Attainment, and Rurality, 2019-2022

Model with education and rurality	Estimate	95% CI, Lower	95% CI, Upper	p-value
**AA and NHPI:**				
Indicator for year 2022	0.0	-3.4	3.5	0.983
Indicator for any college or higher educational	-2.4	-5.5	0.7	0.130
Indicator for rural locations	-18.1	-29.7	-6.5	0.002
Interaction of year and any college or higher educational	2.9	-1.3	7.2	0.179
Interaction of year and rural locations	-3.4	-27.3	20.6	0.783
Interaction of any college or higher educational and rural locations	14.8	0.1	29.4	0.049
Interaction of year, any college or higher educational and rural locations	-4.2	-31.7	23.3	0.762
**Change from 2019 to 2022:**				
Among those with any college or higher education in rural locations	2.2	-10.2	14.6	0.728
Among those with any college or higher education in urban locations	2.8	0.3	5.3	0.030
Among those with high school degree or lower educational in rural locations	-10.7	-32.8	11.4	0.342
Among those with high school degree or lower educational in urban locations	0.4	-3.0	3.8	0.828
Rural-Urban differential change among those with any college degree or higher education	-0.6	-13.1	12.0	0.929
Rural-Urban differential change among those with high school degree or lower education	-11.1	-33.3	11.1	0.328
Higher-lower educational attainment differential change among those in rural locations	12.9	-10.4	36.2	0.277
Higher-lower educational attainment differential change among those in urban locations	2.4	-1.8	6.6	0.266

© 2025 Gidengil C et al. *JAMA Network Open.*

**Black:**				
Indicator for year 2022	3.5	0.6	6.5	0.020
Indicator for any college or higher educational	3.0	-0.1	6.1	0.054
Indicator for rural locations	-6.3	-13.2	0.6	0.076
Interaction of year and education	-0.5	-4.6	3.7	0.829
Interaction of year and rural locations	3.6	-5.1	12.3	0.418
Interaction of education and rural locations	1.7	-9.8	13.2	0.774
Interaction of year, education and rural locations	0.3	-14.9	15.5	0.968
**Change from 2019 to 2022:**				
Among those with any college or higher education in rural locations	8.0	-1.9	17.9	0.114
Among those with any college or higher education in urban locations	3.0	0.2	5.8	0.038
Among those with high school degree or lower educational in rural locations	6.6	-0.6	13.9	0.074
Among those with high school degree or lower educational in urban locations	3.6	0.7	6.6	0.016
Rural-Urban differential change among those with any college degree or higher education	5.0	-5.1	15.0	0.331
Rural-Urban differential change among those with high school degree or lower education	3.0	-4.6	10.6	0.442
Higher-lower educational attainment differential change among those in rural locations	1.4	-8.6	11.3	0.785
Higher-lower educational attainment differential change among those in urban locations	-0.6	-4.7	3.4	0.765
**Hispanic:**				
Indicator for year 2022	3.0	0.6	5.4	0.013
Indicator for any college or higher educational	2.1	-0.6	4.9	0.124
Indicator for rural locations	-4.7	-11.2	1.8	0.158
Interaction of year and education	-3.7	-7.6	0.1	0.058
Interaction of year and rural locations	8.0	-1.5	17.4	0.098

© 2025 Gidengil C et al. *JAMA Network Open.*

Interaction of education and rural locations	-1.7	-12.0	8.7	0.756
Interaction of year, education and rural locations	-7.6	-22.6	7.3	0.315
**Change from 2019 to 2022:**				
Among those with any college or higher education in rural locations	-1.5	-11.1	8.1	0.755
Among those with any college or higher education in urban locations	-0.7	-3.7	2.3	0.668
Among those with high school degree or lower educational in rural locations	11.4	3.0	19.8	0.008
Among those with high school degree or lower educational in urban locations	3.0	0.6	5.3	0.014
Rural-Urban differential change among those with any college degree or higher education	-0.9	-10.6	8.9	0.861
Rural-Urban differential change among those with high school degree or lower education	8.4	-0.2	17.1	0.055
Higher-lower educational attainment differential change among those in rural locations	-12.9	-23.6	-2.2	0.018
Higher-lower educational attainment differential change among those in urban locations	-3.6	-7.4	0.2	0.061
**White:**				
Indicator for year 2022	-1.2	-2.3	-0.2	0.022
Indicator for any college or higher educational	3.0	2.1	3.8	0.000
Indicator for rural locations	-2.4	-3.7	-1.1	0.000
Interaction of year and education	2.7	1.5	3.9	0.000
Interaction of year and rural locations	-2.5	-4.6	-0.4	0.020
Interaction of education and rural locations	-1.8	-3.6	0.0	0.049
Interaction of year, education and rural locations	0.2	-2.5	2.9	0.882
**Change from 2019 to 2022:**				
Among those with any college or higher education in rural locations	-1.4	-2.9	0.0	0.056

© 2025 Gidengil C et al. *JAMA Network Open.*

Among those with any college or higher education in urban locations	1.6	1.0	2.2	0.000
Among those with high school degree or lower educational in rural locations	-3.0	-4.6	-1.3	0.000
Among those with high school degree or lower educational in urban locations	-1.5	-2.5	-0.5	0.004
Rural-Urban differential change among those with any college degree or higher education	-3.1	-4.6	-1.5	0.000
Rural-Urban differential change among those with high school degree or lower education	-1.5	-3.3	0.4	0.120
Higher-lower educational attainment differential change among those in rural locations	1.5	-0.5	3.5	0.134
Higher-lower educational attainment differential change among those in urban locations	3.1	1.9	4.3	0.000

© 2025 Gidengil C et al. *JAMA Network Open.*

eTable 5. Linear Regression Model Estimates Predicting Influenza Immunization with Omnibus Tests for Race and Ethnicity and Its Interactions

**Model without educational attainment or rurality:**					
**Parameters**	**Estimate**	**Standard Error**	**95% CI, Lower**	**95% CI, Upper**	**p-value**
Indicator for year 2022	0.21	0.25	-0.29	0.71	0.415
AA & NHPI	3.51	0.77	2.01	5.02	0.000
Black	-10.79	0.77	-12.30	-9.29	0.000
Hispanic	-4.97	0.65	-6.25	-3.68	0.000
Interaction of year 2022 by:					
AA & NHPI	1.65	1.05	-0.41	3.71	0.118
Black	3.56	1.03	1.54	5.59	0.001
Hispanic	2.04	0.94	0.21	3.88	0.029
**Omnibus test of year by race-and-ethnicity (3 degrees of freedom)**					**0.001**
**Model with educational attainment and without Rurality:**					
**Parameters**	**Estimate**	**Standard Error**	**95% CI, Lower**	**95% CI, Upper**	**p-value**
Indicator for year 2022	-1.97	0.46	-2.86	-1.08	0.000
AA & NHPI	3.51	0.77	1.99	5.02	0.000
Black	-10.48	0.78	-12.01	-8.94	0.000
Hispanic	-4.08	0.68	-5.41	-2.74	0.000
Indicator for any college or higher educational	2.72	0.35	2.04	3.40	0.000
Interaction of year 2022 by:					
AA & NHPI	4.76	1.54	1.73	7.78	0.002
Black	5.63	1.26	3.17	8.10	0.000
Hispanic	5.82	1.15	3.57	8.07	0.000
Any college or higher educational	3.16	0.54	2.10	4.21	0.000
AA & NHPI and any college or higher educational	-4.78	1.59	-7.89	-1.67	0.003
Black and any college or higher educational	-3.02	1.40	-5.76	-0.28	0.031

© 2025 Gidengil C et al. *JAMA Network Open.*

Hispanic and any college or higher educational	-8.05	1.41	-10.80	-5.29	0.000
**Omnibus test of year by race-and-ethnicity (3 degrees of freedom)**					**0.000**
**Omnibus test of year by race-and-ethnicity by any college or higher education (3 degrees of freedom)**					**0.000**
**Model with rurality and without educational attainment:**					
**Parameters**	**Estimate**	**Standard Error**	**95% CI, Lower**	**95% CI, Upper**	**p-value**
Indicator for year 2022	0.67	0.28	0.12	1.21	0.017
AA & NHPI	2.85	0.77	1.34	4.35	0.000
Black	-11.16	0.77	-12.67	-9.66	0.000
Hispanic	-5.45	0.66	-6.74	-4.17	0.000
rural	-4.03	0.44	-4.89	-3.17	0.000
Interaction of year 2022 by:					
AA & NHPI	1.54	1.05	-0.52	3.60	0.143
Black	2.89	1.05	0.83	4.96	0.006
Hispanic	1.24	0.95	-0.63	3.11	0.193
Rural locations	-2.49	0.67	-3.80	-1.18	0.000
AA & NHPI and rural locations	-9.04	5.66	-20.13	2.05	0.110
Black and rural locations	4.29	2.37	-0.36	8.93	0.070
Hispanic and rural locations	6.94	2.90	1.25	12.63	0.017
**Omnibus test of year by race-and-ethnicity (3 degrees of freedom)**					**0.017**
**Omnibus test of year by race-and-ethnicity by location (3 degrees of freedom)**					**0.010**

Data for these models consisted of AANHPI, Black, Hispanic and White respondents, 2019-2022.

Omnibus tests are tests of significance of all three parameters for AANPHI, Black and Hispanic in interaction terms.

© 2025 Gidengil C et al. *JAMA Network Open.*

eTable 6. Sample Sizes, Educational Attainment, and Rural Residence Rates by Race and Ethnicity, Weighted and Unweighted

Column1	Type	Overall	AA & NHPI	Black	Hispanic	White
**2019 Data**						
Overall	Unweighted N	230,720	8,538	19,535	19,436	172,365
	Unweighted Row %	N/A	3.7%	8.5%	8.4%	74.7%
	Weighted Row %	N/A	3.8%	7.8%	6.5%	76.9%
**2022 Data**						
Overall	Unweighted N	285,265	11,349	22,498	20,901	217,423
	Unweighted Row %	N/A	4.0%	7.9%	7.3%	76.2%
	Weighted Row %	N/A	4.5%	8.2%	7.2%	75.4%
**By Education:**						
**2019 Data**						
High School Degree/GED or lower Educational Attainment	Unweighted N	97,930	3,661	11,573	13,101	65,393
	Unweighted Column %	54.5%	54.8%	36.8%	23.7%	60.4%
	Weighted Column %	60.3%	62.9%	48.1%	33.5%	64.0%
Any College or Higher Educational Attainment	Unweighted N	125,751	4,675	7,193	4,606	104,033
	Unweighted Column %	42.4%	42.9%	59.2%	67.4%	37.9%
	Weighted Column %	37.2%	35.3%	48.5%	60.3%	34.3%
Missing	Unweighted N	7,039	202	769	1,729	2,939
	Unweighted Column %	3.1%	2.4%	3.9%	8.9%	1.7%
	Weighted Column %	2.5%	1.8%	3.3%	6.2%	1.7%
**2022 Data**						

© 2025 Gidengil C et al. *JAMA Network Open.*

High School Degree/GED or lower Educational Attainment	Unweighted N	116,956	4,565	12,508	13,443	81,773
	Unweighted Column %	57.0%	57.7%	42.1%	30.6%	61.1%
	Weighted Column %	62.4%	65.8%	52.4%	36.9%	65.8%
Any College or Higher Educational Attainment	Unweighted N	162,517	6,546	9,468	6,387	132,829
	Unweighted Column %	41.0%	40.2%	55.6%	64.3%	37.6%
	Weighted Column %	35.9%	32.4%	45.8%	59.1%	33.0%
Missing	Unweighted N	5,792	238	522	1,071	2,821
	Unweighted Column %	2.0%	2.1%	2.3%	5.1%	1.3%
	Weighted Column %	1.8%	1.7%	1.9%	4.0%	1.2%
**By Rurality:**						
**2019 Data**						
Rural Locations	Unweighted N	41,293	497	1,802	1,196	35,517
	Unweighted Column %	17.9%	5.8%	9.2%	6.2%	20.6%
	Weighted Column %	17.9%	3.4%	10.8%	7.8%	20.0%
Urban Locations	Unweighted N	189,427	8,041	17,733	18,240	136,848
	Unweighted Column %	82.1%	94.2%	90.8%	93.8%	79.4%
	Weighted Column %	82.1%	96.6%	89.2%	92.2%	80.0%
**2022 Data**						
Rural Locations	Unweighted N	52,523	492	2,180	1,508	45,553
	Unweighted Column %	18.4%	4.3%	9.7%	7.2%	21.0%
	Weighted Column %	16.9%	3.1%	9.8%	6.7%	19.4%
Urban Locations	Unweighted N	232,742	10,857	20,318	19,393	171,870
	Unweighted Column %	81.6%	95.7%	90.3%	92.8%	79.0%
	Weighted Column %	83.1%	96.9%	90.2%	93.3%	80.6%

© 2025 Gidengil C et al. *JAMA Network Open.*

Overall N and percentages includes all 7 race-and-ethnicity groups (AI/AN, AA & NHPI, Black, Hispanic, Multiracial, White, and missing); hence row percentages for the 4 largest race-and-ethnicity groups do not add to 100%.
AANHPI = Asian American and Native Hawaiian or other Pacific Islander.

© 2025 Gidengil C et al. *JAMA Network Open.*
