# Supplementary material for: Influenza Vaccination Among People With Medicare by Race and Ethnicity, Education, and Rurality
Source: JAMA Netw Open. 2025 Apr 10;8(4):e254462. doi: 10.1001/jamanetworkopen.2025.4462 (PMC11986769; doi:10.1001/jamanetworkopen.2025.4462)
Supplement: Supplement 2. — Data Sharing Statement [file jamanetwopen-e254462-s002.pdf]

Data Sharing Statement

Gidengil. Influenza Vaccination Among People With Medicare by Race and Ethnicity, Education, and Rurality, 2019-2022. *JAMA Netw Open*. Published April 10, 2025. doi:10.1001/jamanetworkopen.2025.4462

Data
Data available: No

Additional Information
Explanation for why data not available: The data analyzed are not publicly available but are available from the Centers for Medicare & Medicaid Services via a Data Use Agreement.
